# Physical Function in Older Adults

**DOI:** 10.1093/geroni/igaf122.2026

**Published:** 2025-12-31

## Abstract

In this session, research will be presented from bench to bedside to provide insights into functional decline in aging.

